# PPAR *α* contributes to protection against metabolic and inflammatory derangements associated with acute kidney injury in experimental sepsis

**DOI:** 10.14814/phy2.14078

**Published:** 2019-05-18

**Authors:** Takuma Iwaki, Brock G. Bennion, Erin K. Stenson, Jared C. Lynn, Cynthia Otinga, Danijel Djukovic, Daniel Raftery, Lin Fei, Hector R. Wong, W. Conrad Liles, Stephen W. Standage

**Affiliations:** ^1^ Department of Pediatrics University of Washington School of Medicine Seattle Washington; ^2^ Department of Pediatrics University Hospital Faculty of Medicine Kagawa University Kagawa Japan; ^3^ Department of Pathology and Immunology Washington University School of Medicine St. Louis Missouri; ^4^ Department of Pediatrics Section of Critical Care University of Colorado School of Medicine Anschutz Medical Center Children's Hospital Colorado Aurora Colorado; ^5^ Division of Critical Care Medicine Cincinnati Children's Hospital Medical Center Cincinnati Ohio; ^6^ Department of Chemistry and Biochemistry University of Colorado Boulder Colorado; ^7^ Department of Anesthesiology and Pain Medicine University of Washington School of Medicine Seattle Washington; ^8^ Division of Biostatistics and Epidemiology Cincinnati Children's Hospital Medical Center Cincinnati Ohio; ^9^ Department of Pediatrics University of Cincinnati Cincinnati Ohio; ^10^ Department of Medicine University of Washington School of Medicine Seattle Washington

**Keywords:** Acute kidney injury, lipid metabolism, peroxisome proliferator‐activated receptor alpha, sepsis

## Abstract

Sepsis‐associated acute kidney injury (AKI) is a significant problem in critically ill children and adults resulting in increased morbidity and mortality. Fundamental mechanisms contributing to sepsis‐associated AKI are poorly understood. Previous research has demonstrated that peroxisome proliferator‐activated receptor *α* (PPAR *α*) expression is associated with reduced organ system failure in sepsis. Using an experimental model of polymicrobial sepsis, we demonstrate that mice deficient in PPAR *α* have worse kidney function, which is likely related to reduced fatty acid oxidation and increased inflammation. Ultrastructural evaluation with electron microscopy reveals that the proximal convoluted tubule is specifically injured in septic PPAR *α* deficient mice. In this experimental group, serum metabolomic analysis reveals unanticipated metabolic derangements in tryptophan‐kynurenine‐NAD ^+^ and pantothenate pathways. We also show that a subgroup of children with sepsis whose genome‐wide expression profiles are characterized by repression of the PPAR *α* signaling pathway has increased incidence of severe AKI. These findings point toward interesting associations between sepsis‐associated AKI and PPAR *α*‐driven fatty acid metabolism that merit further investigation.

## Introduction

Sepsis is an all‐too‐common, life‐threatening condition that afflicts both old and young (Mayr et al. 2014; Weiss et al. 2015). Sepsis has traditionally been viewed as a syndrome of organ system failure caused by immune dysregulation in the setting of significant infection (Angus and van der Poll 2013; Hotchkiss et al. 2013). Recent findings, however, have pointed to disruption of cellular metabolism as a contributing cause of organ failure in sepsis (Lee and Hüttemann 2014; Singer 2014). The kidney is particularly sensitive to septic insult and acute kidney injury (AKI) is a common complication of sepsis that increases both patient morbidity and mortality (Alobaidi et al. 2015; Fitzgerald et al. 2018).

Our previous research evaluating children with septic shock using genome‐wide expression profiling of circulating leukocytes identified a subpopulation with more severe disease, worse organ system dysfunction, and increased mortality. This cohort of children demonstrated profound suppression of the peroxisome proliferator‐activated receptor alpha (PPAR*α*) signaling pathway (Wong et al. 2009b). PPAR*α* is a ligand‐activated nuclear hormone receptor transcription factor that regulates the expression of genes related to inflammation and cellular lipid metabolism (Wahli and Michalik 2012; Bougarne et al. 2018). Using an experimental model of polymicrobial sepsis, we previously showed that mice lacking PPAR*α* expression (*Ppara* ^−/−^ mice) have decreased survival, consistent with our clinical transcriptomic data (Standage et al. 2012). Furthermore, we recently demonstrated that *Ppara* ^−/−^ mice show evidence of greater cardiac and kidney injury in sepsis than do wild‐type (WT) mice (Standage et al. 2016).

In the current work, we sought to further evaluate the role of PPAR*α* in sepsis‐associated AKI. Using multiple modalities, we show that *Ppara* ^−/−^ mice have inflammatory and metabolic derangement associated with functional renal failure and markers of kidney injury. The proximal convoluted tubule of the nephron is specifically damaged in *Ppara* ^−/−^ mice. Additionally, we report clinical findings that children with septic shock whose genome‐wide expression profiles are characterized by PPAR*α* signaling pathway suppression have greater incidence of severe AKI.

## Materials and Methods

All raw data, images, statistical analysis scripts, and supplementary methods and figures are deposited on the Open Science Framework (https://doi.org/10.17605/osf.io/2jfwx).

### Animal studies

All experiments were approved by the University of Washington Institutional Animal Care and Use Committee. We used 12‐ to 14‐week old male C57Bl/6J mice and age‐matched *Ppara* ^−/−^ mice (B6.129S4‐Pparatm1Gonz/J) raised in our own colonies from breeding pairs purchased from The Jackson Laboratory (Bar Harbor, Maine; Stock Nos: 000664 and 008154, respectively). Sepsis was induced using the cecal ligation and puncture (CLP) model as previously described (Buras et al. 2005; Nemzek et al. 2008; Standage et al. 2012, 2016). Sham‐operated animals underwent the same procedure, including exteriorization and manipulation of their cecum, but the cecum was not ligated or punctured. All mice received postoperative saline, buprenorphine, and imipenem. Because septic mice do not eat postoperatively, chow was removed from all cages, including those of the sham groups to control for the fasted state. For the metabolomics and electron microscopy experiments, cohorts of unoperated, fed, control mice were used to represent healthy baseline. Mice were euthanized and tissue samples were collected 24 h after the operation.

### Blood and urine biomarker assays

Blood was collected by direct cardiac puncture and serum frozen was prepared at −80°C until batch analysis. Serum was subsequently analyzed for blood urea nitrogen (BUN) (Pointe Scientific, Canton, Michigan) and creatinine (Diazyme Laboratories, Poway, California) using enzyme‐based colorimetric assays. Both assays provided values significantly higher than expected norms for mice in all study cohorts. Consistent with multiple prior experiments, the sham mice appeared very well and scored low on our clinical illness severity scoring system (not shown), while the CLP mice were visibly ill. The elevated values were considered an artifact of the analytical system and they are reported here unaltered with the analysis focused on relative differences between groups, assuming a normal baseline in the WT sham cohort.

Urine was collected at 24 h postoperatively using special cages with wire mesh bottoms. Mice were placed singly in urine collection cages at the 12‐h monitoring point until the 24‐h monitoring point. Collected urine was frozen at −80°C until batch analysis. Urine total protein levels were measured using the Pierce BCA assay (Thermo Fisher Scientific, Waltham, Massachusetts).

### Kidney tissue gene expression analysis

Kidneys were collected 24 h after CLP and snap frozen in liquid nitrogen. Total RNA was purified from tissue samples using Trizol reagent (Thermo Fisher Scientific, Waltham, Massachusetts). cDNA was created using a High Capacity cDNA Reverse Transcription Kit and qRT‐PCR was performed using a Sensimix II kit (Bioline, Taunton, Massachusetts) and TaqMan gene expression assays for kidney injury marker 1 (KIM‐1, *Havcr1*), neutrophil gelatinase‐associated lipocalin (NGAL, *Lcn2*), interleukin 6 (*Il6*), IL‐1*β* (*Il1b*), tumor necrosis factor *α* (*Tnfa*), fatty acid translocase (cluster of differentiation 36, *Cd36*), fatty acid transport proteins 1 and 2 (*Slc27a1*,* Slc27a2*), medium chain acyl‐CoA dehydrogenase (*Acadm*), very long‐chain acyl‐CoA dehydrogenase (*Acadvl*), acyl‐coenzyme A oxidase 1 (*Acox1*), acetyl‐CoA carboxylase, carnitine palmitoyltransferase 1a and 2 (*Cpt1a and Cpt2*) and peroxisome proliferator‐activated receptor *α* (*PPARα)* (Thermo Fisher Scientific, Waltham, Massachusetts). Relative gene expression was calculated using the ΔΔ‐Ct method normalizing the results to WT sham condition. On preliminary review of the gene expression data, it became apparent that the PPAR*α* primer/probe set had been contaminated and this target was thus eliminated from final analysis.

### Protein immunoblotting

Total protein was extracted from snap frozen kidney tissue using RIPA buffer and 20 *μ*g loaded onto 4–12% Bis‐Tris gradient gels for protein gel electrophoresis. After transfer, PVDF membranes were stained with Ponceau reagent and imaged to assess protein loading. Washed membranes were subsequently blocked with 5% BSA and then incubated overnight at 4°C in a solution containing both primary antibodies. Rabbit polyclonal anti‐ACADM antibody was obtained from Abcam (ab92461, Lot #: GR15062‐13; Cambridge, Massachusetts). Rabbit polyclonal anti‐ACADVL was obtained from Santa Cruz Biotechnology (sc‐98338, Lot #: A2814; Dallas, Texas). Membranes were subsequently incubated with HRP‐conjugated goat polyclonal anti‐rabbit antibody (Abcam, Lot #: unavailable; ab6721), developed using SuperSignal West Pico Chemiluminescent Substrate (Life Technologies, Carlsbad, California), and imaged on a Bio‐Rad Chemidoc imager (Bio‐Rad, Hercules, California). While neither of our primary antibodies have undergone rigorous validation with knockout controls, their use has been reported in many other studies with findings consistent with specific regulation of their target proteins (Mells et al. 2012; Choi et al. 2014; Tan et al. 2014; Becker et al. 2018; Liu et al. 2018; Stancic et al. 2018; Zheng and Cai 2019).

Relative protein abundance was measured by densitometry using ImageJ (National Institutes of Health, Bethesda, MD). To normalize for protein loading, the band density of target proteins was divided by the density of the total protein Ponceau staining in the region of the membrane at the predicted molecular weight of the protein of interest.

### Electron microscopy

Tissue samples for electron microscopy were obtained at the same time for control and experimental mice under the same conditions. Tissue cut from the renal cortex was fixed with 4% glutaraldehyde in sodium cacodylate buffer, mounted in Epon Araldite epoxy resin blocks, cut by ultramicrotome and visualized on a Hitachi H‐7650 transmission electron microscope. Electron micrographs of 6–9 randomly selected proximal tubular segments were captured for each subject and scored by a blinded reader for evidence of injury. The scoring system focused on specific ultrastructural changes and is given in Table S2.

### Serum metabolomics

Fifty‐microliters of serum underwent methanol extraction and subsequent targeted liquid chromatography–mass spectroscopy. Relative metabolite abundance was quantified by measuring the detected product ion peak area. A detailed description of the metabolomic analysis is provided in the Supplemental Material.

### Assessment of acute kidney injury in critically ill children with septic shock

Acute kidney injury was analyzed retrospectively in a cohort of pediatric patients admitted to intensive care units with a diagnosis of septic shock as part of an ongoing, multicenter genomic and clinical research program. The study was approved by institutional review boards at each participating institution and has been described previously (Shanley et al. 2007; Cvijanovich et al. 2008; Wong et al. 2009b; Basu et al. 2011). Briefly, blood was obtained from each subject within 24 h of admission to the ICU. RNA was extracted from whole blood and genome‐wide expression profiles generated using Affymetrix gene chip technology. Clinical and laboratory data were collected according to each institution's standard of care and 28‐day mortality recorded. All subjects were previously described in studies reporting two endotypes of pediatric septic shock, endotypes A and B, based on the expression patterns of 100 genes (Wong et al. 2011, 2015). The 100 endotyping genes correspond to adaptive immunity, the glucocorticoid receptor signaling pathway, and the PPAR*α* signaling pathway. The majority of these genes is repressed in endotype A subjects, relative to endotype B subjects. Additionally, allocation to endotype A is independently associated with increased odds of persistent multiple organ failure and mortality.

One hundred and ninety‐three subjects with septic shock had both endotyping and clinical data sufficient for AKI staging. Endotype classification was assigned to each subject based on their gene expression profile, as previously described. Diagnosis of AKI was based on the Kidney Disease: Improving Global Outcomes (KDIGO) criteria. Severe AKI was defined as stage 3 (creatinine level ≥ 3 times baseline) and was assessed on day 3 of PICU admission. This cutoff was used because this KDIGO stage has been associated with increased mortality rate in prior studies (Sutherland et al. 2015; Kaddourah et al. 2017). The timing of AKI diagnosis was chosen because: (1) most PICU patients with AKI will develop AKI by day 3, (2) 3 days allow enough time for development of severe AKI, and (3) the passage of time allows for increased creatinine attributable to “prerenal” conditions to improve (Basu et al. 2014).

None of the subjects had baseline, pre‐illness creatinine recorded. Baseline creatinine was therefore estimated using two previously validated different methods. For subjects with recorded height, we estimated baseline creatinine from the calculated body surface area (Zappitelli et al. 2008). For the remaining patients, baseline creatine was estimated with a method accounting for age and gender (Pottel et al. 2008; Hessey et al. 2017).

### Statistical analysis

Statistics were computed using R version 3.5.1 (https://cran.r-project.org). Raw data and analysis scripts are made available on the Open Science Framework (https://doi.org/10.17605/osf.io/2jfwx). As we evaluated the effect of two factors, mouse strain and experimental condition, on outcome indices, we first ran two‐factor analyses of variance (ANOVAs) with an interaction term. If the interaction term was significant, we computed post hoc pairwise comparisons between contrasts of interest defined a priori. If no interaction effect was identified, the two‐factor ANOVA model was repeated without an interaction term to evaluate for main effects. If statistically significant main effects were identified, pairwise comparisons were subsequently made between factor levels. *P* values were adjusted for multiple comparisons using a false discovery rate of 0.05. For the urine protein experiment, the Welch's *t*‐test for unequal variances was used for simple comparisons between two groups. The Fisher exact test was used to compare the fraction of anuirc mice between genotypes. An alpha of 0.05 was used for all statistical tests.

In children with septic shock, we used multivariable logistic regression to evaluate for an association between endotype assignment and AKI. The model included terms to control for severity of illness (PRISM score) and age (Wong et al. 2009b; Stenson et al. 2018).

## Results

### Ppara^−/−^ mice have worse kidney function in experimental polymicrobial sepsis

**Figure 1:**
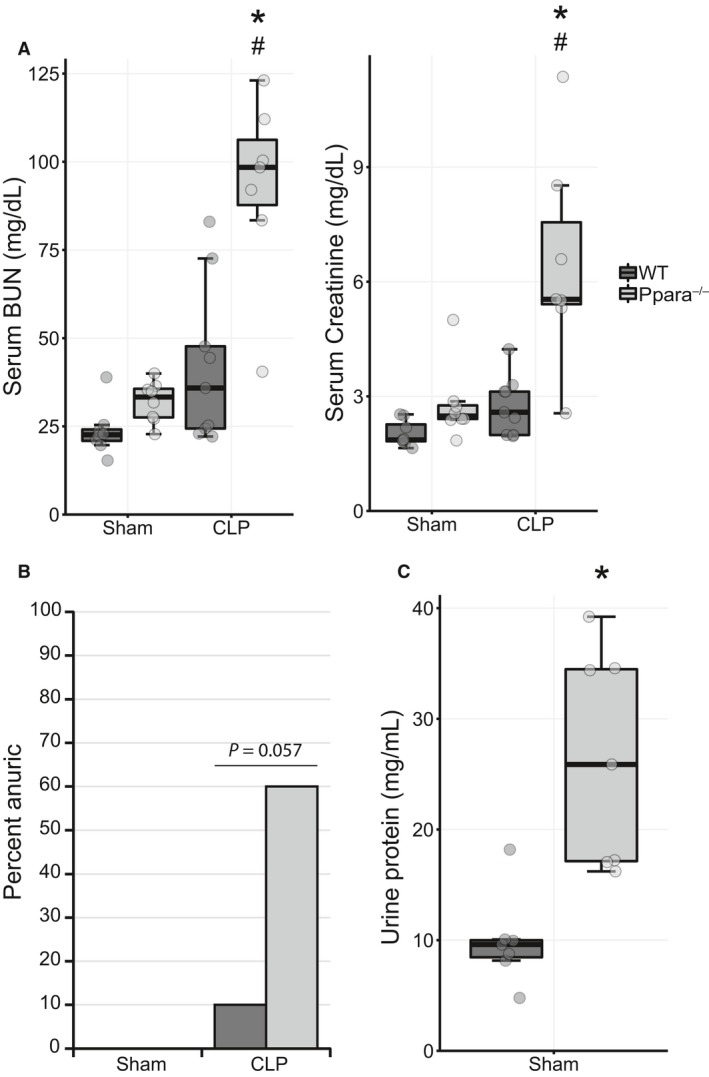
*Ppara* ^−/−^ mice exhibit renal failure in experimental sepsis. 24 h after CLP or sham surgery blood and urine were collected. (A) Serum markers of kidney injury, BUN and creatinine were specifically elevated in *Ppara* ^−/−^ mice. No statistically significant differences were noted between WT and *Ppara* ^−/−^ mice in the sham condition or in WT mice between sham and CLP conditions. Interaction term for a two‐factor ANOVA had a *P* value < 0.01 for both biomarkers and post hoc pairwise comparisons also differed significantly. *P < 0.001 for comparison between strains within condition, ^#^ *P* < 0.001 for comparison between conditions within strain. *n* = 8 WT/Sham; 8, *Ppara* ^−/−^/Sham; 9 WT/CLP; 7, *Ppara* ^−/−^/CLP. (B) Six of 10 septic *Ppara* ^−/−^ mice were anuric when urine was collected between the 12‐ and 24‐h time points. Only 1 of 10 WT mice was anuric after CLP (Fisher exact test, *P* = 0.057). (C) Urine was analyzed for protein only in the sham group due to survivor bias in the CLP cohort. Even with sham surgery, *Ppara* ^−/−^ mice exhibited elevated protein levels in their urine (Welch's *t*‐test, *P* < 0.01). *n* = 7/group.

To investigate the effect of PPAR*α* expression on kidney function in sepsis, WT and *Ppara* ^−/−^ mice underwent CLP to induce polymicrobial sepsis or sham surgery. Serum biomarkers for kidney injury, BUN and creatinine, were higher in *Ppara* ^−/−^ mice, but not WT mice 24 h after CLP, indicating worse renal function in sepsis in the *Ppara* ^−/−^ cohort (Fig. 1A). Although the sham operation represents a minor injury and there appeared to be a slight elevation in BUN and creatinine levels in the *Ppara* ^−/−^ sham group, no statistically significant differences were noted between WT and *Ppara* ^−/−^ mice in the sham condition. Likewise, the minor trends in biomarker elevation in the WT mice between sham and CLP conditions were not significant (Table 1).

Measurement of urine protein as a marker of kidney injury was complicated by a large proportion of anuric *Ppara* ^−/−^ mice. The majority of septic *Ppara* ^−/−^ mice had no urine output between 12 and 24 h after CLP, indicating very poor kidney function, while only one WT mouse was anuric (Fig. 1B). No sham mice were anuric. When assessed in the sham cohorts, urine protein was elevated in *Ppara* ^−/−^ compared to WT mice (Fig. 1C). This biomarker was not analyzed in the CLP cohorts due to concern for significant survivor bias.

### Tissue mRNA expression of markers of kidney injury and inflammation are more elevated in septic Ppara^−/−^ mice

**Figure 2:**
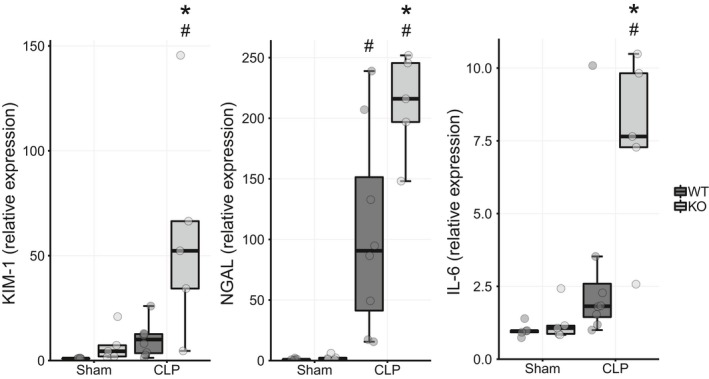
Tissue markers of injury and inflammation are increased in *Ppara* ^−/−^ mice in sepsis. mRNA expression of renal injury markers KIM‐1 and NGAL as well as the inflammatory marker IL‐6 was more elevated in *Ppara* ^−/−^ mice than WT mice 24 h after CLP. No statistically significant differences were noted in KIM‐1 and IL‐6 expression between WT and *Ppara* ^−/−^ mice in the sham condition or in WT mice between sham and CLP conditions. *P < 0.01 for comparison between strains within condition, ^#^ *P* < 0.01 for comparison between conditions within strain. *n* = 5 WT/Sham; 5 *Ppara* ^−/−^/Sham; 8 WT/CLP; 5, *Ppara* ^−/−^/CLP.

To assess direct tissue injury, we measured kidney mRNA expression of KIM‐1 and NGAL, which are elevated in renal failure, and the inflammatory markers IL‐6, IL‐1*β*, and TNF*α*. Kidney KIM‐1, NGAL, and IL‐6 expression was dramatically increased in CLP operated *Ppara* ^−/−^ mice compared with the sham condition. WT CLP mice only showed elevation in NGAL expression, which was still lower than the level observed in *Ppara* ^−/−^ mice (Fig. 2). The slight upward trends in WT KIM‐1 and IL‐6 expression did not reach statistical significance. TNF*α* and IL‐1*β* had no interaction effect between mouse strain and experimental condition, but main effects for condition were identified in both, indicating increased expression in the CLP over the sham condition irrespective of genotype (Fig. S1).

### Expression of enzymes related to transport and oxidation of fatty acids is lower in Ppara^−/−^ kidneys

**Figure 3:**
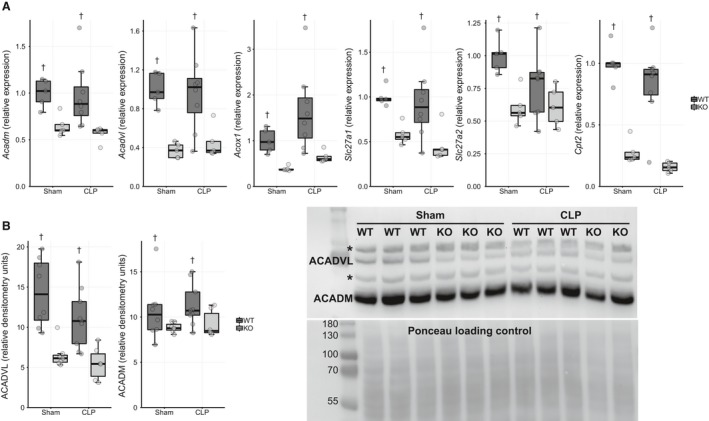
Tissue expression of enzymes related to fatty acid transport and oxidation are lower in *Ppara* ^−/−^ mice. (A) mRNA expression of genes associated with lipid metabolism was lower in *Ppara* ^−/−^ mice than WT irrespective of condition. No interaction effect was noted on two‐factor ANOVA, but main effect for strain was significant (^†^ *P* < 0.05). *n* = 5 WT/Sham; 5 *Ppara* ^−/−^/Sham; 8 WT/CLP; 5 *Ppara* ^−/−^/CLP. (B) Protein levels of ACADM and ACADVL, two enzymes involved with FAO, were lower in *Ppara* ^−/−^ mice. Again, only main effects for strain were observed in the ANOVA model (^†^ *P* < 0.05). * indicates nonspecific bands. A representative western blot is displayed. All immunoblot images are available in online data repository. *n* = 8 WT/Sham; 7 *Ppara* ^−/−^/Sham; 9 WT/CLP; 6 *Ppara* ^−/−^/CLP.

Because the renal cortex relies primarily on lipid substrate for energy provision (Weidemann and Krebs 1969), we evaluated expression of genes related fatty acid metabolism. Gene expression of enzymes associated with fatty acid oxidation (FAO) (*Acadm*,* Acadvl*, and *Acox1*) and fatty acid transport (*Slc27a1*,* Slc27a2, Cpt2*) was lower in *Ppara* ^−/−^ kidney tissue than in WT kidney in both sham and CLP conditions (Fig. 3A). Expression levels did not differ between conditions within each genotype. Kidney expression of fatty acid translocase (*Cd36*) was, however, higher in the CLP mice than in the sham without difference between genotypes. No differences were observed in the expression of *Cpt1a*, the primary transporter of fatty acids into the mitochondria, between either strain or condition (Figure S2). Consistent with their gene expression, protein levels of ACADM and ACADVL were lower in the *Ppara* ^−/−^ kidney (Fig. 3B).

### Transmission electron microscopy reveals more severe ultrastructural damage in Ppara^−/−^ proximal tubules

**Figure 4:**
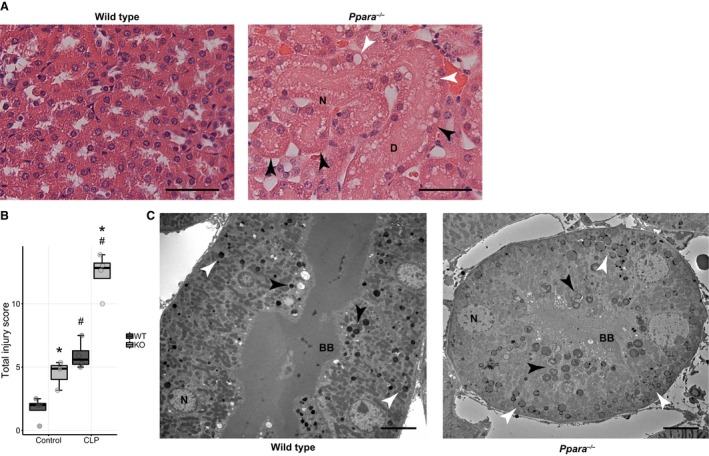
Septic *Ppara* ^−/−^ mice exhibit more damage to the proximal tubule. (A) Representative H and E stained sections of renal cortex at 20× magnification. *Ppara* ^−/−^ tubules show dilatation (D), epithelial flattening (black arrow heads), vacuolization (white arrow heads), and nuclear drop out (N). Scale bar = 50 *μ*m. (B) Formal scoring on ultrastructural evaluation of proximal tubular segments demonstrates worse injury in *Ppara* ^−/−^ mice. Interaction term in two‐factor ANOVA significant (*P* = 0.00011) with subsequent post hoc pairwise contrasts (*P < 0.05 for comparison between strains within condition, ^#^ *P* < 0.05 for comparison between conditions within strain). (C) Representative electron micrographs in longitudinal and transverse cross‐section show brush border (BB) disruption, increased autophagosome, and autolysosome abundance (black arrow heads), and greater lipid droplet number and size (white arrow heads) in the *Ppara* ^−/−^ mice. Nucleus (N). 800 × magnification. Scale bar = 8 *μ*m. All scored electron micrographs are available in online data repository. *n* = 4 WT/Control; 3 *Ppara* ^−/−^/Control; 4 WT/CLP; 4, *Ppara* ^−/−^/CLP.

We previously reported that *Ppara* ^−/−^ mice had worse kidney histological injury scores on evaluation by light microscopy (Standage et al. 2016). Specifically, we observed multifocal areas of tubular degeneration and necrosis in *Ppara* ^−/−^ kidneys, characterized by tubular dilatation with epithelial flattening, cytoplasmic vacuolization, and nuclear dropout (Fig. 4A). These findings, coupled with a recognition that the *Ppara* ^−/−^ proximal tubule would likely be specifically vulnerable to injury because of its high reliance on FAO, motivated an analysis of proximal tubular ultrastructure using electron microscopy.

The total proximal tubule injury score was higher for *Ppara* ^−/−^ mice after CLP than WT (Fig. 4B). *Ppara* ^−/−^ proximal tubular epithelial cells had far greater disruption of their brush border, more numerous endocytic vesicles, and dramatically more abundant autophagosomes (Fig. S3). Regarding lysosome abundance, we observed main effects for both strain and condition, but no interaction between them. *Ppara* ^−/−^ mice have lipid droplets in their proximal tubular epithelial cells in the control condition that do not change in abundance with sepsis. Control WT mice, however, demonstrate no lipid droplets, but develop them after CLP in equivalent abundance to the *Ppara* ^−/−^ mice. We neither saw differences in the loss of basal interdigitating processes nor any changes in mitochondrial structure or integrity (Fig. S3). Visualized glomeruli and distal convoluted tubule segments appeared normal.

### Serum metabolomic analysis reveals evidence of renal failure and metabolic derangement in Ppara^−/−^ mice

**Figure 5:**
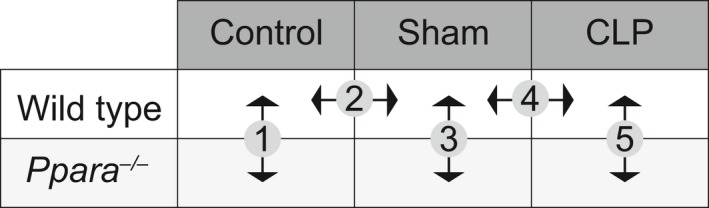
Serum metabolomics analysis comparisons. Serum metabolite levels were analyzed in all three conditions for both genotypes to evaluate for important metabolic differences. Five direct comparisons were made to highlight contrasts of greatest interest. *n* = 10 WT/Control; 10 *Ppara* ^−/−^/Control; 10 WT/Sham; 5 *Ppara* ^−/−^/Sham; 6 WT/CLP; 8 *Ppara* ^−/−^/CLP.

The kidney plays a central role in regulating the metabolic milieu in both health and stress states. We therefore undertook a serum metabolomic analysis of *Ppara* ^−/−^ and WT mice in control, sham, and CLP conditions 24 h after surgery. Sham mice were fasted to replicate the self‐imposed anorexic state exhibited by septic mice. Rather than evaluate every possible comparison individually, we focused our analysis on five important contrasts that would illuminate metabolic differences between the control, sham, and CLP conditions in WT mice and show how *Ppara* ^−/−^ mice differed from WT in each condition (Fig. 5). Pathway analysis for each comparison was subsequently performed using MetaboAnalyst (http://www.metaboanalyst.ca/) (Xia and Wishart 2016; Chong et al. 2018).

#### Comparison 1

**Figure 6:**
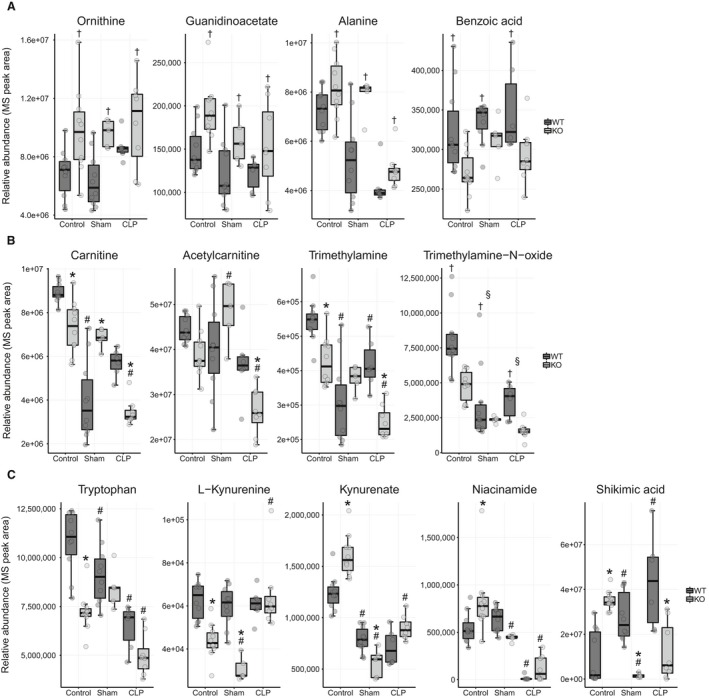
Metabolites that differ between WT and *Ppara* ^−/−^ mice in the control condition and across all conditions (Comparison 1). (A) Metabolites associated with the urea cycle and nitrogen excretion differ at baseline between WT and *Ppara* ^−/−^ mice. (B) Carnitine and associated quaternary ammonium compounds are lower in *Ppara* ^−/−^ mice. (C) *Ppara* ^−/−^ mice show derangement in metabolites associated with the tryptophan‐kynurenine‐NAD+ pathway. See Figure S4A for calculated fold change and *P* values. *P < 0.05 for comparison between strains within condition, ^#^ *P* < 0.05 for comparison between previous condition within strain, ^†^ *P* < 0.05 for main effect only for strain across all conditions, and ^§^ *P* < 0.05 for main effect only for comparison with previous condition.

Metabolites that differed between *Ppara* ^−/−^ and WT mice in the control condition and across all conditions (main effect for strain) related to three important biological processes associated with renal function and energy metabolism. First, serum levels of metabolites associated with the urea cycle (ornithine, guanidinoacetate, and alanine) were elevated in *Ppara* ^−/−^ versus WT mice (Fig. 6A). Additionally, benzoic acid, which is involved with excretion of nitrogenous waste, was lower in *Ppara* ^−/−^ mice. This indicates a higher degree of protein turn over in *Ppara* ^−/−^ mice, which is consistent with previous findings demonstrating that PPAR*α* activation suppresses amino acid metabolism and attendant urea cycle function (Tremblay and Qureshi 1993; Feoli‐Fonseca et al. 1996; Kersten et al. 2001; Makowski et al. 2009; Brosnan and Brosnan 2010; Kersten 2014).

Second, carnitine is lower in the serum of *Ppara* ^−/−^ mice. Carnitine is necessary for fatty acid transport and oxidation (Fig. 6B). Its production, which is primarily localized to the kidney (Rebouche and Engel 1980; Guder and Wagner 1990), is enhanced by PPAR*α* transcriptional regulation (Makowski et al. 2009; Ringseis et al. 2012). Other quaternary ammonium compounds, structurally similar to carnitine (trimethylamine and trimethylamine‐N‐oxide) were also lower in *Ppara* ^−/−^ mice.

Third, multiple metabolites associated with the tryptophan‐kynurenine‐NAD^+^ pathway (tryptophan, L‐kynurenine, kynurenate, niacinamide, and shikimate) differed between WT and *Ppara* ^−/−^ mice (Fig. 6C). This pathway subserves many biological functions, but importantly, NAD^+^ production is its primary output (Cervenka et al. 2017). NAD^+^ is an essential energy carrier for many bioenergetic reactions. MetaboAnalyst pathway analysis identified tryptophan metabolism as the most dysregulated pathway in this comparison (Fig. S4).

#### Comparison 2

**Figure 7:**
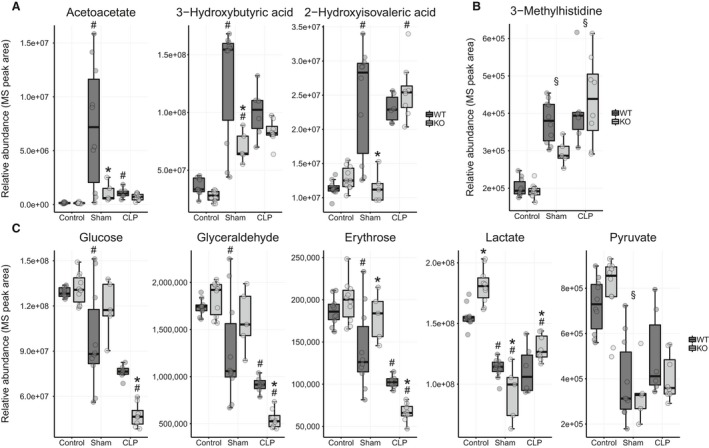
Metabolites that differ between control and sham conditions in WT mice (Comparison 2). (A) Ketone metabolites are increased in the fasted sham state. *Ppara* ^−/−^ mice have lower ketone levels. (B) 3‐methylhistidine, a marker for muscle breakdown is also elevated in the sham condition in both strains. (C) Metabolites associated with carbohydrate metabolism are decreased and are lower in *Ppara* ^−/−^ mice in sepsis. See Figure S5A for calculated fold change and *P* values. *P < 0.05 for comparison between strains within condition, ^#^ *P* < 0.05 for comparison between previous condition within strain, and ^§^ *P* < 0.05 for main effect only for comparison with previous condition.

Metabolic signatures characteristic of fasting distinguished control and sham conditions in WT mice. Serum ketones (acetoacetate, 3‐hydroxybutyric acid, and 2‐hydroxyisovaleric acid) were dramatically upregulated (Fig. 7A). Levels of 3‐methylhistidine, a marker of skeletal muscle breakdown (Long et al. 1981; Stortz et al. 2018), were increased in the sham animals indicating the mobilization of amino acids from muscle (Fig. 7B). Levels of several gluconeogenic (alanine, arginine, proline, methionine, tryptophan, and tyrosine) and ketogenic (lysine, tryptophan, and tyrosine) amino acids were reduced, likely due to consumption in the fasted state. Metabolites associated with carbohydrate metabolism (glucose, glyceraldehyde, erythrose, lactate, and pyruvate) were decreased (Fig. 7C). MetaboAnalyst identified pathways related to carbohydrate, amino acid, and ketone body metabolism as the most different between control and sham states in WT mice (Fig. S5).

#### Comparison 3

The primary difference between *Ppara* ^−/−^ and WT mice in the sham condition is that ketone abundance was lower in *Ppara* ^−/−^ mice (Fig. 7A, Fig. S6). We observed this previously (Standage et al. 2017) and attribute the difference to decreased FAO, which is needed to generate ketones (Kersten et al. 1999; Leone et al. 1999).

#### Comparison 4

**Figure 8:**
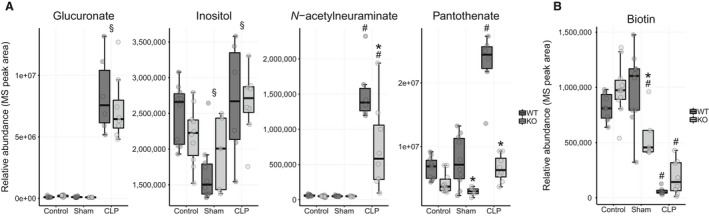
Metabolites that differ between sham and CLP conditions in WT mice (Comparison 4). (A) Metabolites associated with stress responses are elevated in the CLP over the sham condition. *Ppara* ^−/−^ mice do not increase N‐acetylneuraminate or pantothenate abundance like WT mice. (B) Biotin is decreased in sepsis in both genotypes. See Figure S7A for calculated fold change and *P* values. *P < 0.05 for comparison between strains within condition, ^#^ *P* < 0.05 for comparison between previous condition within strain, and ^§^ *P* < 0.05 for main effect only for comparison with previous condition.

Two important features characterize the metabolic differences between the sham and CLP conditions in WT mice. First, metabolites associated with stress responses dramatically increased (Fig. 8A). Serum glucuronate levels rose nearly 160‐fold in the CLP condition and inositol, its immediate biosynthetic precursor, was also increased. Glucuronidation is a host defense mechanism that eliminates endogenous toxins, environmental chemicals, and reactive metabolites (Guillemette 2003; Kalthoff et al. 2010). Levels of N‐acetylneuraminate, a monosaccharide involved with protein and lipid glycosylation, were profoundly elevated in sepsis as well. Significant alterations in protein sialyation have been reported in sepsis and systemic inflammation with several studies demonstrating cleavage of sialyl residues from proteins in these states (Piagnerelli et al. 2005; Gornik et al. 2007, 2011; Novokmet et al. 2014). Additionally, pantothenate levels increased dramatically after CLP.

Second, metabolic changes potentially associated with decreased energy production were evident. Septic mice had lower levels of tryptophan and niacinamide, metabolites essential to NAD^+^ biosynthesis (Fig. 6C), and low biotin, which is an enzymatic cofactor for mitochondrial carboxylation reactions (Fig. 8B). We also noted reduced levels of the ketone acetoacetate compared with the sham condition, which we and others have previously observed in sepsis (Fig. 7A) (Lanza‐Jacoby et al. 1990; Standage et al. 2017).

Metaboanalyst evaluation identified pathways related to glucuronate and inositol metabolism, pantothenate and coenzyme A metabolism, and the TCA cycle as differentially regulated between sham and CLP conditions (Fig. S7).

#### Comparison 5

**Figure 9:**
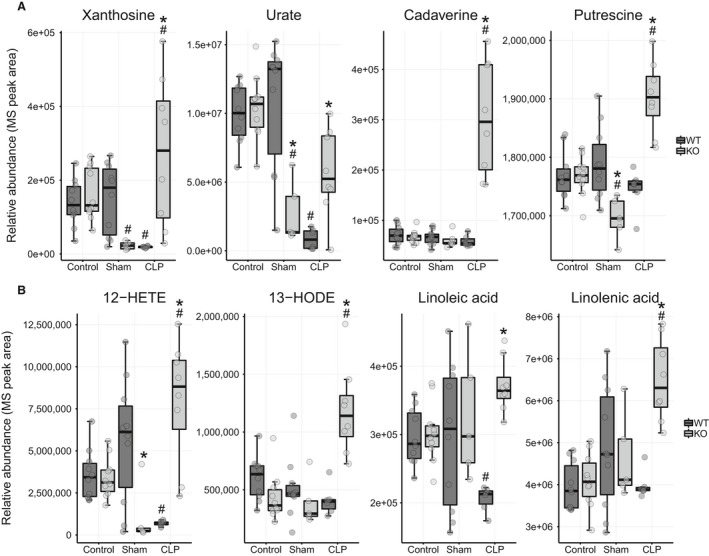
Metabolites that differ between WT and *Ppara* ^−/−^ mice in the CLP condition (Comparison 5). (A) Metabolites associated with renal failure are elevated in *Ppara* ^−/−^ over WT mice in sepsis. (B) *Ppara* ^−/−^ mice have much higher levels of inflammatory oxygenated lipids and their precursors than WT mice in sepsis. See Figure 10A for calculated fold change and *P* values. *P < 0.05 for comparison between strains within condition, ^#^ *P* < 0.05 for comparison between previous condition within strain.

Metabolite differences between WT and *Ppara* ^−/−^ mice in the septic condition provide further evidence of renal function impairment, exacerbated inflammation, and metabolic derangement. Xanthosine, urate, and the polyamines, cadaverine and putrescine, were considerably elevated in the *Ppara* ^−/−^ mice and are associated with kidney failure (Fig. 9A) (Campbell et al. 1978; Saito et al. 1983; Igarashi et al. 2006; Rhee et al. 2010; Goek et al. 2013; Zhang et al. 2016; Hocher and Adamski 2017; Hussain et al. 2017; Srivastava et al. 2018). Oxidized lipids mediate inflammatory signaling and are associated with kidney injury (Rhee et al. 2010). The abundance of two of these lipoxygenase products (12‐HETE and 13‐HODE) were dramatically elevated in the *Ppara* ^−/−^ mice along with their polyunsaturated fatty acid precursors (linoleic and linolenic acid; Fig. 9B). Xanthosine, glucose‐1‐phosphate, urate, and adenylosuccinate are all metabolites involved with nucleotide biosynthesis, degradation, and salvage pathways (Nelson and Cox 2012) and might represent increased cell death as nucleic acid metabolites are released for recycling.

Carbohydrate metabolites decreased following CLP in the serum of *Ppara* ^−/−^ versus WT mice (Fig. 7C) indicating increased cellular reliance on glucose as well as impaired gluconeogenic capacity in the absence of sufficient FAO. Regarding other possible metabolic stress responses observed in WT mice, although *Ppara* ^−/−^ mice augmented glucoronate levels similar to WT mice, they did not increase N‐acetylneuraminate to the same degree, nor do they raise serum pantothenate levels (Fig. 8A). Shikimic acid, a precursor of tryptophan biosynthesis related to NAD^+^ metabolism, was also lower in *Ppara* ^−/−^ mice (Fig. 6C).

**Figure 10:**
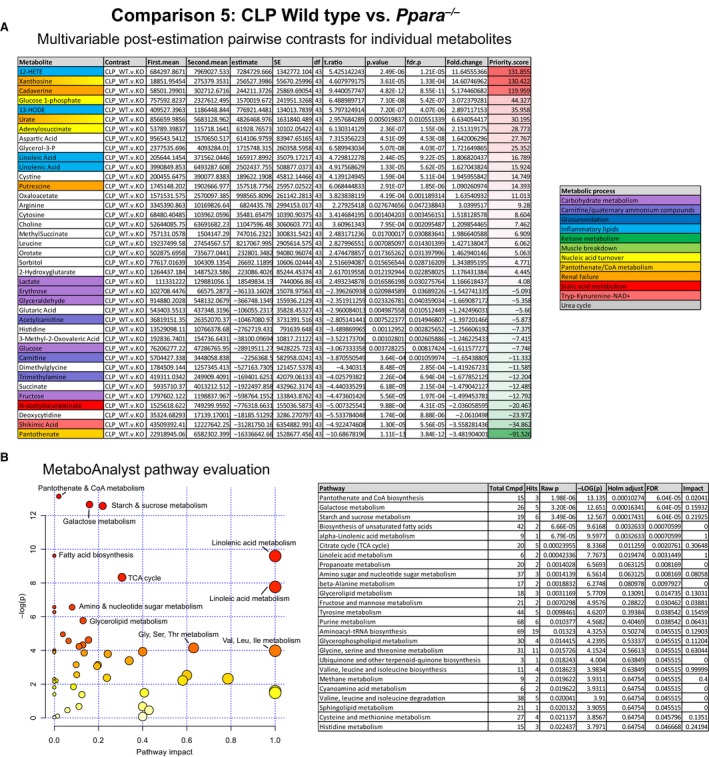
Lists of differentially abundant metabolites and altered metabolic pathways between WT and *Ppara* ^−/−^ mice in sepsis. (A) In the CLP condition, *Ppara* ^−/−^ serum metabolic profile is characterized by increased levels of metabolites associated with renal failure, inflammatory lipids, and nucleic acid turnover. Additionally, *Ppara* ^−/−^ mice had lower levels of pantothenate and N‐acetylneuraminate, metabolites associated with stress responses, as well as decreased amounts of metabolites involved with tryptophan, carbohydrate, and lipid metabolism. This list was generated by analyzing differences between strain and condition using two‐factor ANOVA for each metabolite. Pairwise, post‐estimation contrasts were made for each metabolite that had a significant interaction term. *P* values for these comparisons were adjusted using an FDR of 0.5 and only the significantly different metabolites were included in the list. Metabolites were ranked using a priority score calculated as the product of the fold change and negative log of the *P* value. (B) Pathway analysis using MetaboAnalyst revealed that pathways involving pantothenate, carbohydrate, TCA, and fatty acid metabolism as most different between WT and *Ppara* ^−/−^ mice in sepsis. Only significantly altered pathways are included in the list.

Pathway analysis identified metabolic processes associated with pantothenate and CoA biosynthesis, carbohydrate and lipid metabolism as the most discrepant in this comparison (Fig. 10).

### Septic children with genome‐wide expression profiles characterized by PPAR*α* suppression exhibit more kidney injury

Using unsupervised genome‐wide expression profiling, we previously identified two endotypes of septic shock in children admitted to the pediatric ICU (Cvijanovich et al. 2008; Wong et al. 2009a,2009b). Children with endotype A were found to have higher severity of illness (PRISM‐III scoring), greater number of organ system failures, and decreased survival compared to endotype B. A notable feature of endotype A is repression of genes corresponding to the PPAR*α* signaling pathway (Standage et al. 2012).

We assessed whether children with septic shock classified as endotype A by their genome‐wide expression profiles demonstrated more AKI than children classified under endotype B. Development or persistence of stage III AKI (severe) by day 3 of septic shock was the primary analysis outcome. One hundred and ninety‐three subjects had both endotype and day 3 creatinine data, which we used to stage AKI. None of the subjects had baseline kidney disease. Thirty‐three (17%) had stage three AKI on day 3 of septic shock. As shown in the Table 1, after accounting for baseline illness severity (PRISM‐III score) and age, assignment to endotype A was independently associated with increased odds of stage 3 AKI (OR: 2.50, *P* = 0.04).

**Table 1:** Children with septic shock endotype characterized by PPAR*α* suppression are at greater risk of severe AKI

Variable	OR	95% CI	*P* value
Endotype	2.50	1.04–6.0	0.04
PRISM‐III	1.12	1.07–1.17	<0.001
Age	0.99	0.86–1.13	0.85

Multivariable logistic regression testing for the association between endotype outcome variable and stage 3 AKI on day 3 of septic shock. Adjusted for PRISM‐III score and age.

## Discussion

Using a model of experimental polymicrobial sepsis, we have shown that *Ppara* ^−/−^ mice develop AKI while their WT counterparts do not. Serum BUN and creatinine levels are increased in septic *Ppara* ^−/−^ mice and a far greater proportion of *Ppara* ^−/−^ mice become anuric after CLP. Even with the mild insult of sham operation, *Ppara* ^−/−^ mice have elevated urinary protein levels, which point to an underlying vulnerability to AKI.

Gene expression markers of injury (KIM‐1 and NGAL) and inflammation (IL‐6) are specifically upregulated in the *Ppara* ^−/−^ kidney. Our finding that mRNA expression levels of many lipid transporters and FAO enzymes are decreased in *Ppara* ^−/−^ mice are consistent with the known regulatory influence of PPAR*α* on those genes and findings from previous studies (Feingold et al. 2008; Rakhshandehroo et al. 2010). Protein levels of two FAO enzymes, ACADM and ACADVL, were also lower in the *Ppara* ^−/−^ kidney tissue indicating that translational regulation of these pathways is commensurate with their transcriptional regulation.

Ultrastructural changes noted with electron microscopy in the proximal tubular epithelial cells clearly demonstrate evidence of decreased FAO and worse injury in the septic *Ppara* ^−/−^ kidney. The presence of lipid droplets in the *Ppara* ^−/−^ proximal tubules in both control and CLP conditions indicates a constitutively low level of FAO that potentiates sepsis‐associated injury because the proximal tubule specifically is so highly reliant on FAO for ATP production (Weidemann and Krebs 1969; Bobulescu 2010). This finding is consistent with the observed lower expression of lipid transporters and FAO enzymes in that strain. Interestingly, WT mice do not have lipid droplets in the control condition, but develop them after CLP despite demonstrating no changes in FAO enzyme expression and maintaining adequate renal function. Although WT mice do not show a statistically significant increase in IL‐6, expression of IL‐1*β* and TNF*α* is elevated in the CLP condition for both WT and *Ppara* ^−/−^ mice. Inflammation suppresses FAO in the kidney, which has been well described in multiple renal injury models (Johnson et al. 2005; Zager et al. 2005; Feingold et al. 2008; Takahashi et al. 2011). Metabolic pathway regulation can occur at the transcriptional, translational, and posttranslational levels and metabolic pathway flux may be altered without seeing changes in enzyme expression. We propose that a threshold effect may be at play in the development of lipid droplets and AKI in our model. Control WT mice do not have lipid droplets because they have normal levels of FAO. *Ppara* ^−/−^ mice have lower levels of FAO and manifest lipid droplets at baseline without significant evidence of renal dysfunction. Sepsis‐induced inflammation suppresses FAO in both strains. While the relative degree of this suppression only results in the development of lipid droplets in WT mice, in *Ppara* ^−/−^ mice that start with lower levels of FAO, proximal tubules develop critical energy limitations that compromise functional capacity.

Loss of brush border architecture observed in *Ppara* ^−/−^ mice indicates failure of the absorptive and excretory functions of the proximal tubule essential to renal function. Dramatically increased abundance of autophagosomes points toward the presence two potential cellular pathologies addressed by autophagy: (1) damaged and dysfunctional organelles in need of recycling, (2) energy depletion (Kroemer et al. 2010). This second problem is likely especially active in the *Ppara* ^−/−^ mice that cannot meet increased ATP demands by FAO. They may therefore undertake autophagy to provide an alternative energy supply. Sunahara et al. (2018) recently showed that WT C57/B6 mice subjected to CLP‐induced sepsis had increased autophagy in renal tubular epithelial cells 6–8 h after surgery, that decreased toward sham‐operated levels at 24 h. Our WT mice had only a mild elevation in the qualitative abundance in autophagosomes at 24 h, which could be consistent with Sunahara et al., but we did not evaluate our animals at the 6–8 h time point. Autophagy has been shown to be augmented in the liver by PPAR*α* signaling (Jiao et al. 2014; Lee et al. 2014), but no reports have previously evaluated the relationship between autophagy and PPAR*α* in the kidney. Augmentation of autophagy in our *Ppara* ^−/−^ mice would necessarily be mediated by other signaling pathways.

The most salient finding from our metabolomic study in septic mice is that metabolites associated with renal failure and inflammation have significantly increased abundance in the *Ppara* ^−/−^ cohort. This is consistent with other results reported in this manuscript: serum and urine markers of kidney function as well as tissue expression of genes activated in renal injury and inflammation. PPAR*α* is well known to impose a generally anti‐inflammatory influence on the immune response (Wahli and Michalik 2012; Bougarne et al. 2018). It is therefore not surprising that tissue and circulating mediators of inflammation are higher in *Ppara* ^−/−^ mice with sepsis. Accompanying these observations are multiple signatures of metabolic dysfunction in the *Ppara* ^−/−^ mice.

The metabolomics analysis recapitulated our previous findings that *Ppara* ^−/−^ mice fail to mobilize ketones in the fasted state and that they have lower circulating levels of carbohydrate substrate (Standage et al. 2017). *Ppara* ^−/−^ mice also have elevation of metabolites associated with the urea cycle across all conditions, indicating increased reliance on amino acids for energy production, a dependence that also imposes an increased excretory burden on the kidney to eliminate attendant nitrogenous wastes. They also demonstrate lower levels of carnitine necessary for transport and oxidation of fatty acids, which is expected due to the dependency of that pathway on PPAR*α* regulation.

We detected unanticipated evidence of metabolic defects in the *Ppara* ^−/−^ mice in the tryptophan‐kynurenine‐NAD^+^ and pantothenate pathways, both of which are central to cellular metabolism. Over 95% of tryptophan in the body is metabolized to NAD^+^ for utilization in bioenergetic processes (Cervenka et al. 2017). This pathway flows through various kynurenine compounds which have been shown to be dysregulated in inflammation and sepsis (Chen and Guillemin 2009; Changsirivathanathamrong et al. 2011; Mangge et al. 2014) as well as in chronic kidney disease (Goek et al. 2013; Hocher and Adamski 2017). Recent, important work has shown that kidney NAD^+^ biosynthesis is directly related to FAO and renal injury in model organisms and human studies (Tran et al. 2016; Poyan Mehr et al. 2018). Furthermore, PPAR*α* regulates enzymes in the tryptophan‐kynurenine‐NAD^+^ biosynthetic pathway, including quinolinate phosphoribosyltransferase, which performs the final step of NAD^+^ synthesis (Shin et al. 1999; Agudelo et al. 2014). PPAR*α* may therefore have an important role in cellular energy provision beyond merely maintaining FAO.

The significant elevation of pantothenate levels in septic WT mice and their lack of change in *Ppara* ^−/−^ mice are another potentially important feature. Pantothenate is an essential nutrient incorporated into the coenzyme A molecule, which serves as an indispensable cofactor for many central metabolic reactions (Leonardi et al. 2005). In states of physiologic stress, coenzyme A is hydrolyzed to produce pantetheine, which is further cleaved by vanin enzymes to yield pantothenate and cysteamine, which has antioxidant and cytoprotective effects (Pitari et al. 2000; Berruyer et al. 2004; Naquet et al. 2014). PPAR*α* regulates the vanin pantetheinase enzyme (Rommelaere et al. 2013; van Diepen et al. 2014). Increased pantothenate abundance could possibly represent a stress response mechanism involving pantetheine cleavage from coenzyme A to produce cysteamine that *Ppara* ^−/−^ mice lack.

The clinical relevance of these findings is highlighted by our analysis of critically ill children with septic shock. We saw that children categorized according to genome‐wide expression profiles as sepsis endotype A, which is characterized by profound suppression of the PPAR*α* signaling pathway, had much greater odds of severe AKI than children assigned to endotype B. To our knowledge similar findings have not previously been reported.

We readily acknowledge some limitations of this work. First, animal models do not recapitulate perfectly human disease (Seok et al. 2013; Osuchowski et al. 2014; Takao and Miyakawa 2015). We have taken care, however, to make our model clinically relevant by targeting a WT mortality of about 20–25% at 7 days and administering fluids, antibiotics, and analgesics (Standage et al. 2016). Another limitation is that we utilized whole body *Ppara* ^−/−^ mice and we have shown previously that these mice have cardiac dysfunction and dysregulation serum levels of metabolic substrate (Standage et al. 2017). It is plausible to attribute poor renal function in *Ppara* ^−/−^ mice to poor cardiac function. To address this question, we are currently developing a tissue‐specific *Ppara* ^−/−^ mouse. Despite this possibility, other kidney injury models not dependent on cardiac function have shown that PPAR*α* has direct importance in preventing AKI (Portilla et al. 2006; Li et al. 2012, 2013). Furthermore, our EM analysis showed specific proximal tubular injury in the *Ppara* ^−/−^ kidney and it is known that the epithelial cells that comprise this tubule are highly reliant on FAO for energy provision (Weidemann and Krebs 1969). Another limitation is that we did not directly demonstrate lower FAO in the *Ppara* ^−/−^ kidney or the CLP condition, but rather inferred it from our gene and protein expression experiments and several published reports that show reduction of FAO in the *Ppara* ^−/−^ kidney and in the injured WT kidney (Portilla 2003; Johnson et al. 2005; Zager et al. 2005; Tanaka et al. 2011; Li et al. 2012; Simon and Hertig 2015). In future studies using kidney‐specific PPAR*α* deletion, we will directly quantify renal cortical lipid levels and rates of FAO. Finally, our clinical findings in children with septic shock are retrospective in nature. The data analyzed, however, were collected prospectively as part of a large, ongoing, multicenter clinical/translational investigation, thus minimizing potential bias.

In conclusion, we have shown an association between PPAR*α* expression and sepsis‐associated AKI in our experimental model. The mechanism whereby PPAR*α* could contribute to maintenance of renal function likely relies on augmentation of FAO and other metabolic pathways necessary for cellular energy provision and by reduction of tissue and circulating inflammatory mediators. Our corroborative clinical data indicate that similar PPAR*α* dependent processes may be operative in human sepsis. Future investigation is necessary to confirm these findings and elucidate rigorously the underlying mechanism.

## Conflict of Interest

HRW holds advisory board appointments for Eccrine Systems, Endpoint Health, and Inflammatix and holds sepsis biomarker related patents. No other conflict of interest, financial or otherwise is declared by the authors.

## Supporting information




**Data S1.** Serum metabolomics.
**Figure S1.** Tissue markers of inflammation are increased in sepsis. mRNA expression of the inflammatory markers IL‐1β and TNFα was elevated in CLP operated mice over sham operated mice 24 h after surgery.
**Figure S2.** Tissue expression of *Cd36* and *Cpt1a*. mRNA expression of *Cd36* differed significantly only between conditions (main effect alone, ^§^ *P* < 0.05).
**Figure S3.** Individual components of proximal tubule injury score are worse in septic *Ppara* ^*−/−*^ mice. A: Two‐factor ANOVA models had significant interaction terms for brush border, endocytic vesicle, autophagosome and lipid droplet components of the injury score.
**Figure S4.** Lists of differentially abundant metabolites and altered metabolic pathways between WT and *Ppara* ^*−/−*^ mice in the control condition.
**Figure S5.** Lists of differentially abundant metabolites and altered metabolic pathways between control and sham conditions in WT mice.
**Figure S6.** Lists of differentially abundant metabolites and altered metabolic pathways between WT and *Ppara* ^*−/−*^ mice in the sham condition.
**Figure S7.** Lists of differentially abundant metabolites and altered metabolic pathways between sham and CLP conditions in WT mice.
**Figure S8.** PCA analysis and outlier removal from metabolomics data set.
**Table S1.** Liquid chromatography gradient conditions.
**Table S2.** Scoring system for rating proximal tubular injury on electron micrographs.
