# Supplementary material for: PPAR α contributes to protection against metabolic and inflammatory derangements associated with acute kidney injury in experimental sepsis
Source: Physiol Rep. 2019 May 18;7(10):e14078. doi: 10.14814/phy2.14078 (PMC6525329; doi:10.14814/phy2.14078)
Supplement: Supplementary file 1 — Data S1. Serum metabolomics. Figure S1. Tissue markers of inflammation are increased in sepsis. mRNA expression of the inflammatory markers IL‐1β and TNFα was elevated in CLP operated mice over sham operated mice 24 h after surgery. Figure S2. Tissue expression of Cd36 and Cpt1a. mRNA expression of Cd36 differed significantly only between conditions (main effect alone, § P < 0.05). Figure S3. Individual components of proximal tubule injury score are worse in septic Ppara −/− mice. A: Two‐factor ANOVA models had significant interaction terms for brush border, endocytic vesicle, autophagosome and lipid droplet components of the injury score. Figure S4. Lists of differentially abundant metabolites and altered metabolic pathways between WT and Ppara −/− mice in the control condition. Figure S5. Lists of differentially abundant metabolites and altered metabolic pathways between control and sham conditions in WT mice. Figure S6. Lists of differentially abundant metabolites and altered metabolic pathways between WT and Ppara −/− mice in the sham condition. Figure S7. Lists of differentially abundant metabolites and altered metabolic pathways between sham and CLP conditions in WT mice. Figure S8. PCA analysis and outlier removal from metabolomics data set. Table S1. Liquid chromatography gradient conditions. Table S2. Scoring system for rating proximal tubular injury on electron micrographs. [file PHY2-7-e14078-s001.docx]

**Supplemental Methods**

Serum metabolomics

*Reagents:* Acetonitrile, ammonium acetate, methanol and acetic acid (LC-MS grade) were all purchased from Fisher Scientific (Pittsburgh, Pennsylvania). The standard compounds corresponding to the targeted metabolites were purchased from Sigma-Aldrich (Saint Louis, Missouri) and Fisher Scientific (Pittsburgh, Pennsylvania). Stable isotope-labeled tyrosine (L-tyrosine-^13^C_2_), glutamic acid (L-glutamate-^13^C_2_), lactate (L-lactate-^13^C_3_) and glucose (glucose-^13^C_6_) standards were purchased from Cambridge Isotope Laboratories, Inc. (Tewksbury, Massachusetts). The purities of non-labeled standards were >95-99% whereas the purities of the four ^13^C labeled compounds were > 99%.

*Sample Preparation:* Frozen serum samples were thawed at room temperature for 45 minutes, and 50 µL of each sample was transferred into a 2 mL Eppendorf vial (Fisher Scientific, Pittsburgh, Pennsylvania). Protein precipitation and metabolite extraction was performed by adding 150 µL of methanol containing L-glutamate-^13^C_2_ and glucose-^13^C_6_ standards (these two stable-isotope labeled standards were added to methanol in order to monitor sample prep), and then the mixture was vortexed for 1 minute and stored at -20°C for 20 minutes. Afterwards, the samples were centrifuged at 14000 rpm for 10 minutes, 150 µL of the supernatants were collected into new Eppendorf vials and dried using a Vacufuge Plus evaporator (Eppendorf, Hauppauge, New York). The dried samples were stored at -20°C, and prior to the targeted LC-MS analysis were reconstituted in 500 µL of 10 mM ammonium acetate in 30% water/70% acetonitrile + 0.2% acetic acid containing L-tyrosine-^13^C_2_ and L-lactate-^13^C_3_ standards (these two standards were added to the reconstituting solvent in order to monitor LC-MS system performance). The samples were filtered through 0.45 µm PVDF filters (Phenomenex, Torrance, California) prior to LC-MS analysis

*Liquid Chromatography-Mass Spectrometry (LC-MS) Conditions:* Targeted HILIC liquid chromatography (LC)-mass spectrometry (MS) metabolite analysis of plasma samples was performed at The Northwest Metabolomics Research Center at the University of Washington, Seattle following previously described LC-MS protocol (1–4).

The LC system was composed of two Agilent 1260 binary pumps, an Agilent 1260 auto-sampler and Agilent 1290 column compartment containing a column-switching valve (Agilent Technologies, Santa Clara, California). Each sample was injected twice, 10 µL for analysis using negative ionization mode and 2 µL for analysis using positive ionization mode. Both chromatographic separations were performed in HILIC mode on two parallel Waters XBridge BEH Amide columns (150 x 2.1 mm, 2.5 µm particle size, Waters Corporation, Milford, Massachusetts). While one column was performing the separation, the other column was getting reconditioned and ready for the next injection. The flow rate was 0.300 mL/min, auto-sampler temperature was kept at 4 ̊C, the column compartment was set at 40 ̊C, and total separation time for each ionization mode was 20 min. The mobile phase was composed of Solvents A (10 mM ammonium acetate in 90% H2O /5% acetonitrile/5% methanol + 0.2% acetic acid) and B (10 mM ammonium acetate in 10%H2O/5%methanol/85% acetonitrile + 0.2% acetic acid). The gradient conditions for both separations were identical and are shown in Supplemental Table 1.

After the chromatographic separation, MS ionization and data acquisition was performed using AB Sciex QTrap 5500 mass spectrometer (AB Sciex, Toronto, Ontario, Canada) equipped with electrospray ionization (ESI) source and operating in multiple-reaction-monitoring (MRM) mode. The source and collision gas was N_2_ (99.999% purity). The ESI source conditions were: Curtain Gas (CUR) = 25 psi, Collision Gas (CAD) = high, Ion Spray Voltage (IS) = ±3.8KV, Temperature (TEM) = 500° C, Ion Source Gas 1 (GS1) = 50 psi and Ion Source Gas 2 (GS2) = 40 psi. 78 and 119 metabolites belonging to all major metabolic pathways were targeted in positive and negative ionization modes, respectively. In addition, 4 MRMs corresponding to the four stable isotope-labeled internal standards were monitored (two in each ESI mode). 120 metabolites and 4 isotope-labeled internal standards were measured across all the study samples. Metabolites were measured as relative MRM peak areas. In order to monitor the LC-MS assay performance and to assess the reproducibility of the measurements, a quality control (QC) serum sample was injected per every 10 study samples. The LC-MS assay was run over 2-day period of non-stop data acquisition. 121 metabolites and 4 isotope-labeled internal standards were measured in the QC sample. The average CV over the 2-day period was 6.6% (based on MRM peak areas without any MS signal normalization) and > 90% of measured MRM peaks had CV < 10%.

The extracted MRM peaks were integrated using AB Sciex MultiQuant 2.1 software and exported to an Excel spreadsheet for further data analysis.

Detailed statistical analysis

Data from animals noted to have surgical complications or anatomic abnormalities (e.g. lacerated bowel, cystic kidney) were removed from the analyses.

*Metabolomics analysis:* Raw metabolite data were read into the statistics analysis program. Missing data for each metabolite were deemed to be below the threshold of detection for the system and were replaced with one half the lowest value obtained for that metabolite. Preliminary box plots were generated for each metabolite to assess the variance of the data. It was determined that most data were generally normal in their distribution and that no transformations would be necessary prior to analysis. A few metabolites, however, had very abnormal distributions of their metabolites (putrescine, hippuric acid, and margaric acid). On close evaluation of the data, it became evident that the last 14 samples on the mass spectroscopy run had abnormally elevated levels of putrescine. Because the sample order had been randomized prior to mass spectroscopy measurement, we were able to normalize the elevated values across conditions prior to further analysis. We were unable to determine the source of variation nor correct the widely discrepant values for hippuric acid and margaric acid, so those metabolites were eliminated from the analysis. Additionally, a single dramatically elevated sucrose value was deleted.

This refined data set was uploaded to the MetaboAnalyst Time-series/Two-factor tool for three-dimensional PCA analysis to identify group outliers. We noted overall that the experimental groups clustered fairly well with a few notable exceptions (Supplemental Figure 8). Three sham *Ppara^-/-^* mice had localized to the CLP dimensional space indicating that they had developed a more severe, sepsis-like condition. A few other subjects were distant from their group clusters as well. To maintain group integrity for this exploratory analysis, these were identified for removal from the final data set that would undergo definitive analysis. The multivariate analysis approach that we employed is documented explicitly in the R script provided with the raw data.

The data were then prepared for submission to the MetaboAnalyst Pathway Analysis tool. Because this tool can only analyze a single factor, the refined data set was split into five pairwise comparisons that correlated with the comparisons employed in our multivariate analysis. Each data set was individually uploaded. Missing value estimation was skipped because we had previously performed that ourselves. No further sample normalization, data transformation, or data scaling were used. We selected the Mus musculus (KEGG) pathway library and submitted the analysis accepting the default parameters.

**Supplemental References**

1. **Buas MF**, **Gu H**, **Djukovic D**, **Zhu J**, **Onstad L**, **Reid BJ**, **Raftery D**, **Vaughan TL**. Candidate serum metabolite biomarkers for differentiating gastroesophageal reflux disease, Barrett’s esophagus, and high-grade dysplasia/esophageal adenocarcinoma. *Metabolomics Off J Metabolomic Soc* 13, 2017.

2. **Lohavanichbutr P**, **Zhang Y**, **Wang P**, **Gu H**, **Nagana Gowda GA**, **Djukovic D**, **Buas MF**, **Raftery D**, **Chen C**. Salivary metabolite profiling distinguishes patients with oral cavity squamous cell carcinoma from normal controls. *PloS One* 13: e0204249, 2018.

3. **Miles FL**, **Navarro SL**, **Schwarz Y**, **Gu H**, **Djukovic D**, **Randolph TW**, **Shojaie A**, **Kratz M**, **Hullar MAJ**, **Lampe PD**, **Neuhouser ML**, **Raftery D**, **Lampe JW**. Plasma metabolite abundances are associated with urinary enterolactone excretion in healthy participants on controlled diets. *Food Funct* 8: 3209–3218, 2017.

4. **Parent BA**, **Seaton M**, **Djukovic D**, **Gu H**, **Wheelock B**, **Navarro SL**, **Raftery D**, **O’Keefe GE**. Parenteral and enteral nutrition in surgical critical care: Plasma metabolomics demonstrates divergent effects on nitrogen, fatty-acid, ribonucleotide, and oxidative metabolism. *J Trauma Acute Care Surg* 82: 704–713, 2017.

**Supplemental Figures**

Supplemental Figure 1. Tissue markers of inflammation are increased in sepsis. mRNA expression of the inflammatory markers IL-1β and TNFα was elevated in CLP operated mice over sham operated mice 24 hours after surgery. No interaction effect was noted on 2-factor ANOVA, but main effect for condition was significant (§ p < 0.05).


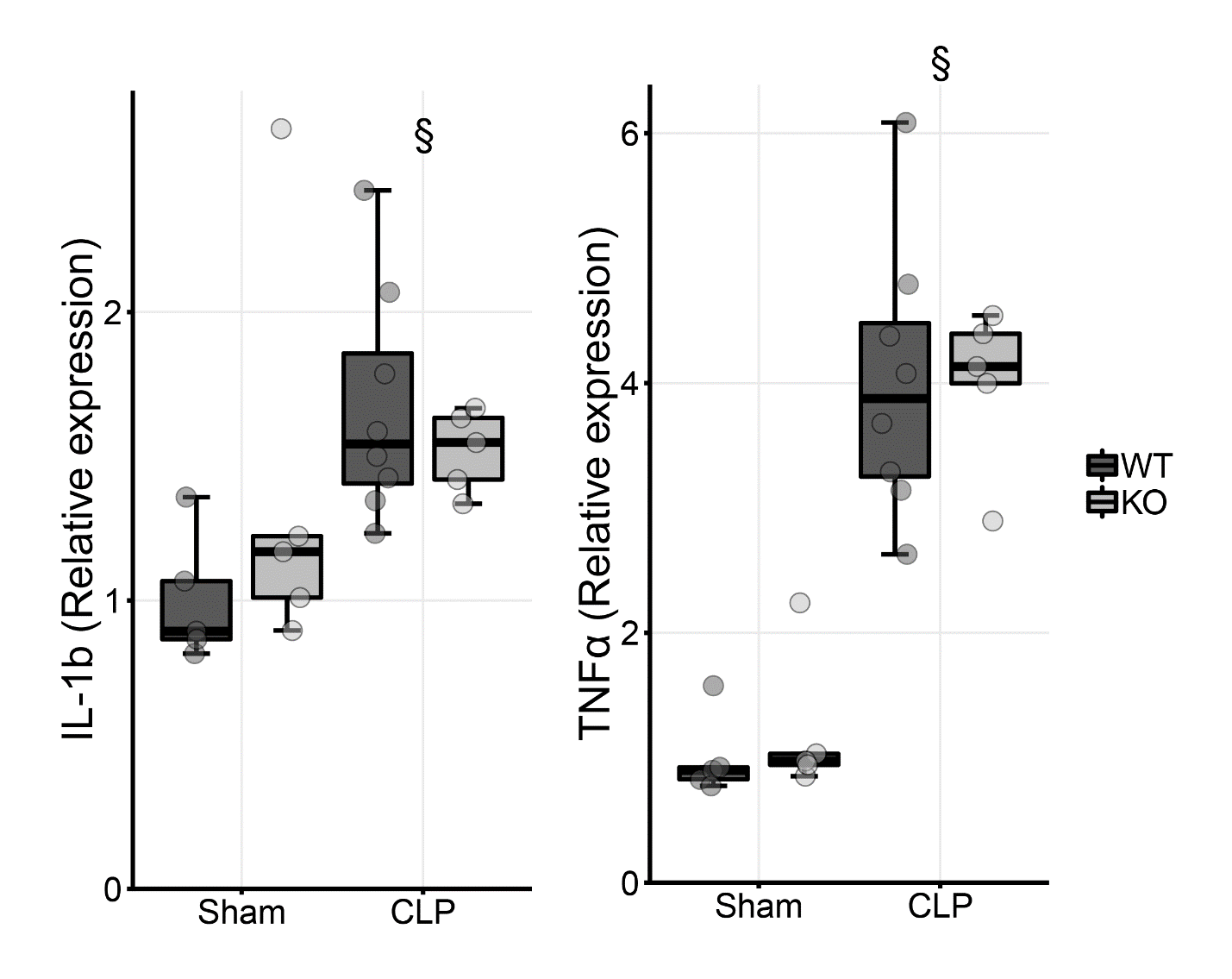


Supplemental Figure 2. Tissue expression of *Cd36* and *Cpt1a*. mRNA expression of *Cd36* differed significantly only between conditions (main effect alone, § p < 0.05). No differences were observed in *Cpt1a* expression.


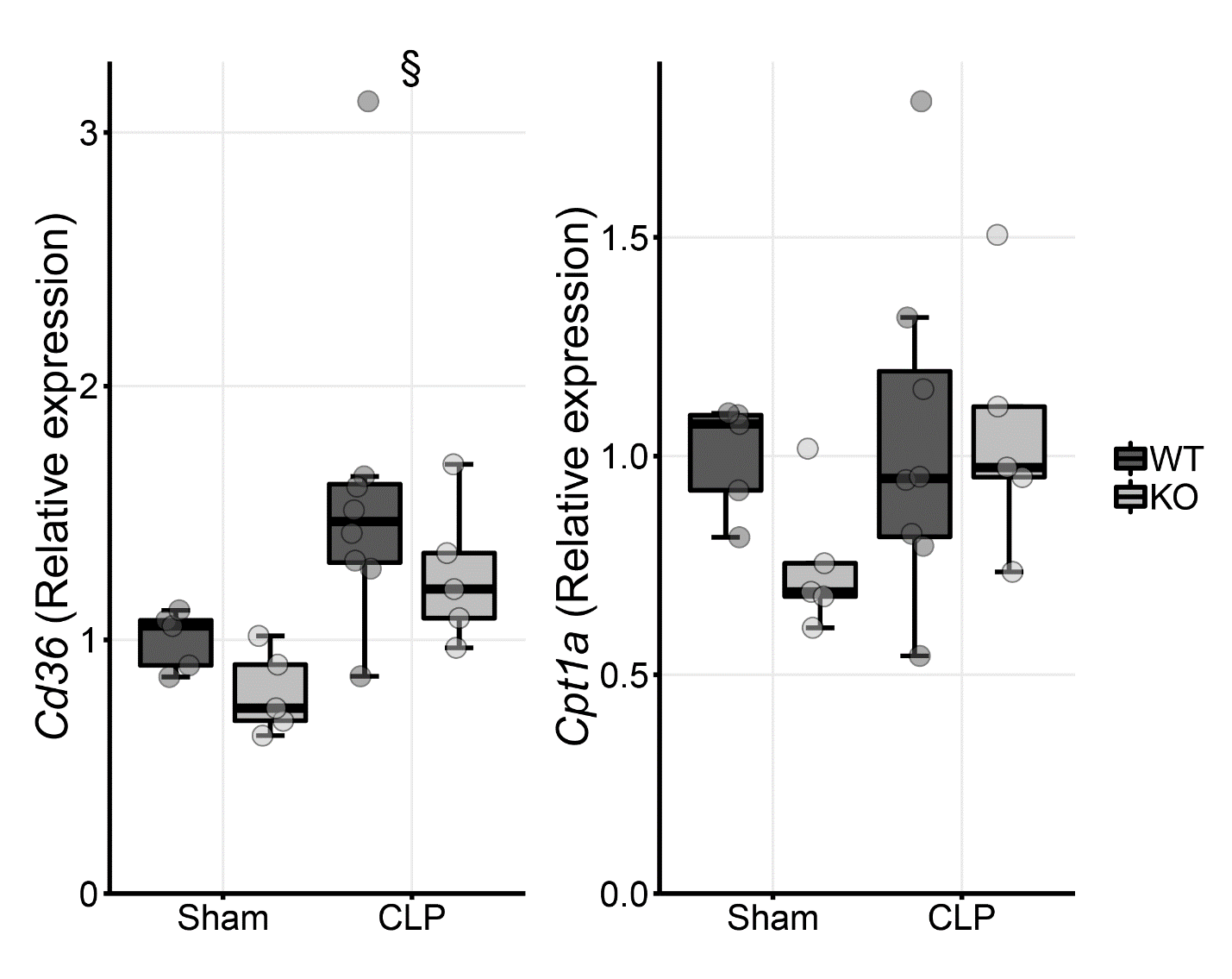


Supplemental Figure 3. Individual components of proximal tubule injury score are worse in septic *Ppara^-/-^* mice. A: 2-factor ANOVA models had significant interaction terms for brush border, endocytic vesicle, autophagosome and lipid droplet components of the injury score. Post-hoc pairwise contrast significance is noted (* p < 0.05 for comparison between strains within condition, # p < 0.05 for comparison between conditions within strain). The lysosome component had main effects for both terms, but no interaction (§ p < 0.05 for condition, † p < 0.05 for strain, n.s. = nonsignificant). B: Representative electron micrographs show significant brush border (BB) disruption and abundant, complex autophagosomes (black arrow heads) in the *Ppara^-/-^* mice. WT mice have few prominent endocytic vesicles (EV), but demonstrate primarily simple lysosomes (black arrow heads). Few lipid droplets (white arrow heads) are present in these sections. Nucleus (N). 1800x magnification. Scale bar = 4 μm.


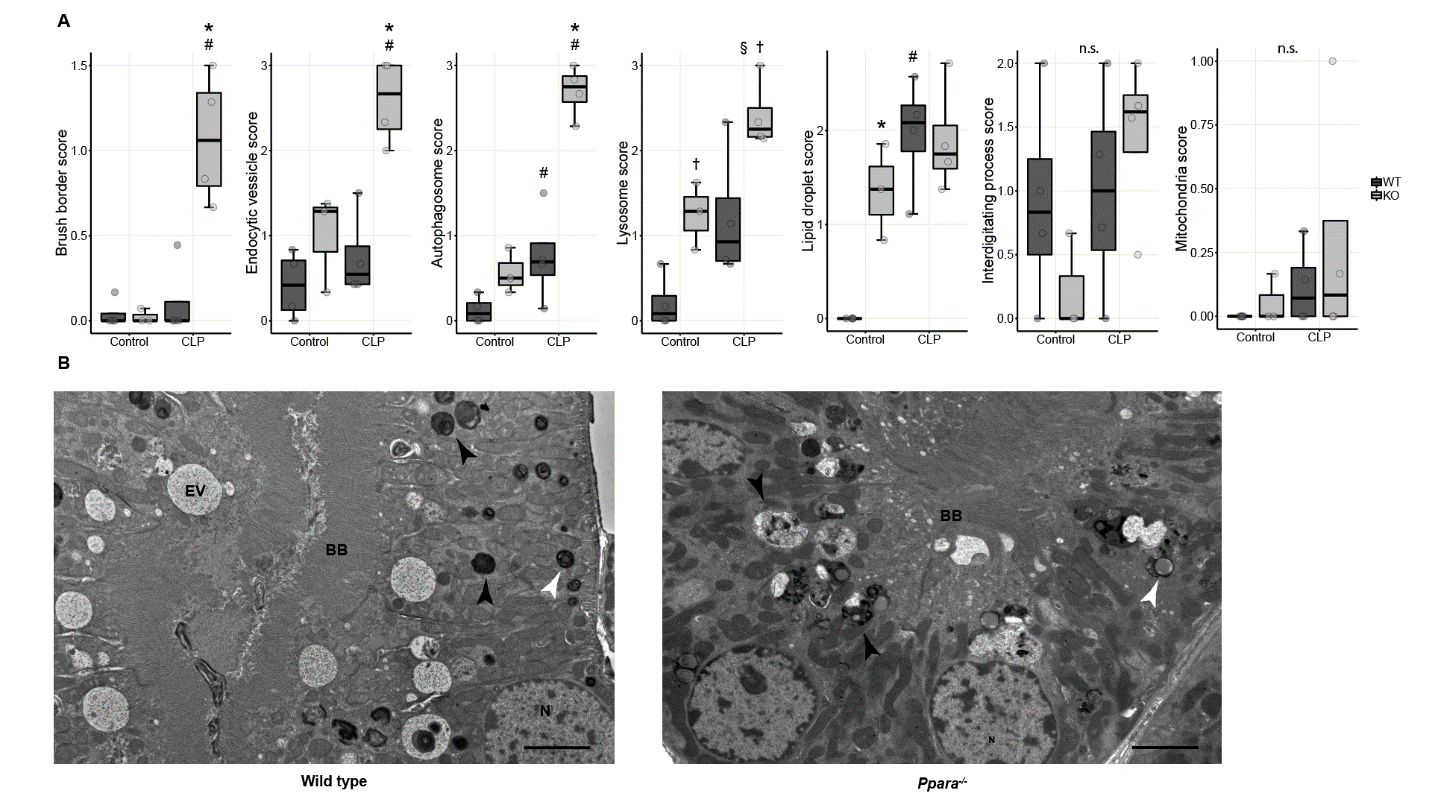


Supplemental Figure 4. Lists of differentially abundant metabolites and altered metabolic pathways between WT and *Ppara^-/-^* mice in the control condition. A: *Ppara^-/-^* mouse serum metabolite levels show evidence of increased urea cycle activity, decreased carnitine abundance, and dysregulation of the tryptophan-kynurenine-NAD^+^ pathway at baseline. This list was generated by analyzing differences between strain and condition using 2-factor ANOVA for each metabolite. Pairwise, post-estimation contrasts were made for each metabolite that had a significant interaction term. P values for these comparisons were adjusted using an FDR of 0.5 and only the significantly different metabolites were included in the list. Metabolites were ranked using a priority score calculated as the product of the fold change and negative log of the p value. B: Pathway analysis using MetaboAnalyst revealed that pathways involving tryptophan, carbohydrate metabolites, ketones, and multiple amino acids differed between WT and *Ppara^-/-^* mice. Only significantly altered pathways are included in the list.


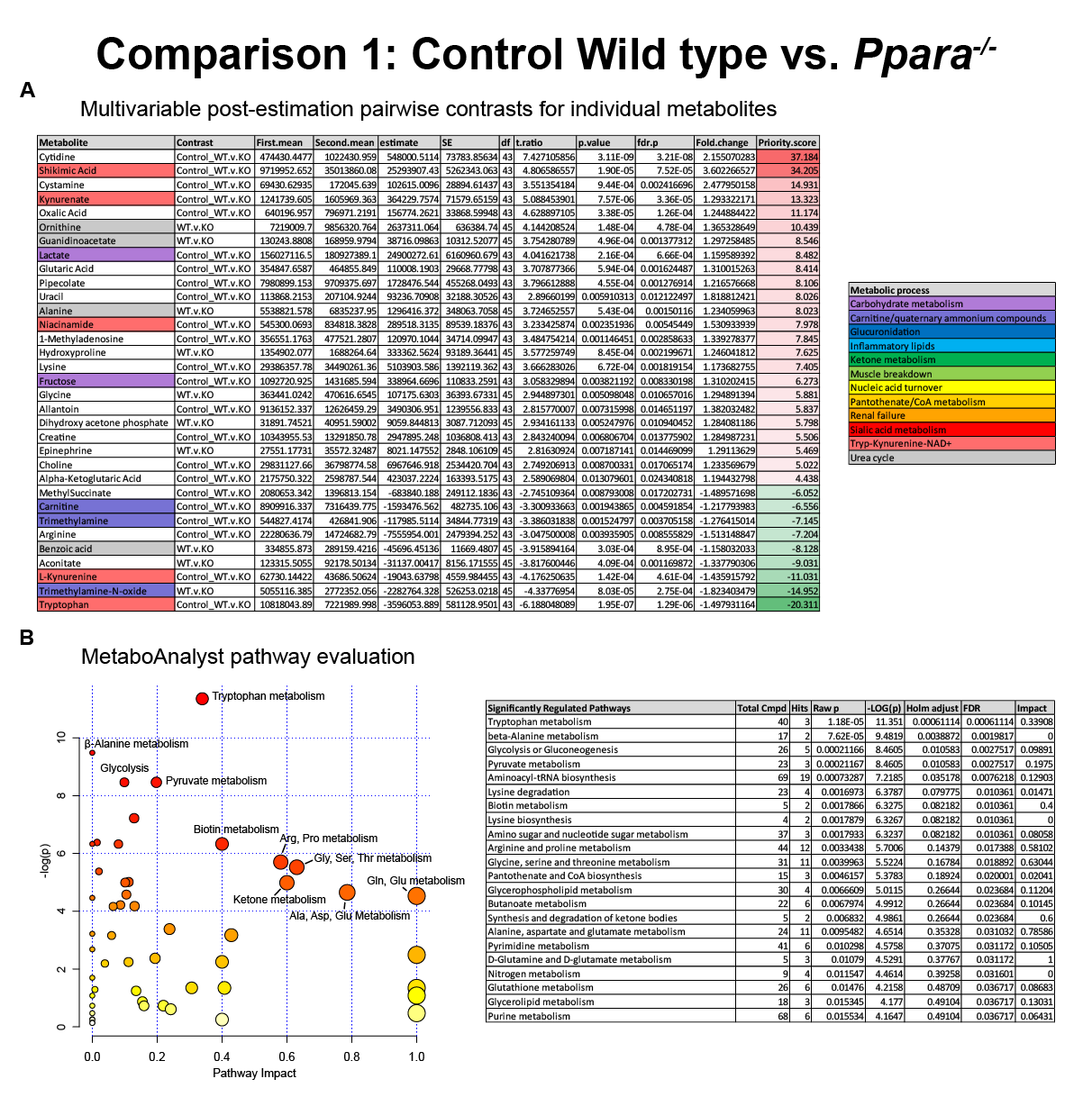


Supplemental Figure 5. Lists of differentially abundant metabolites and altered metabolic pathways between control and sham conditions in WT mice. A: Sham WT mice show significant elevations in ketone metabolites and in 3-methylhistidine, a marker of muscle breakdown. Carbohydrate metabolites are generally decreased as is carnitine and its related compounds. These changes are indicative of the fasting state of these animals, which results in carbohydrate and fatty acid consumption and ketogenesis. B: Pathway analysis using MetaboAnalyst revealed that pathways primarily involving carbohydrate and ketone metabolism differed between control and sham conditions.


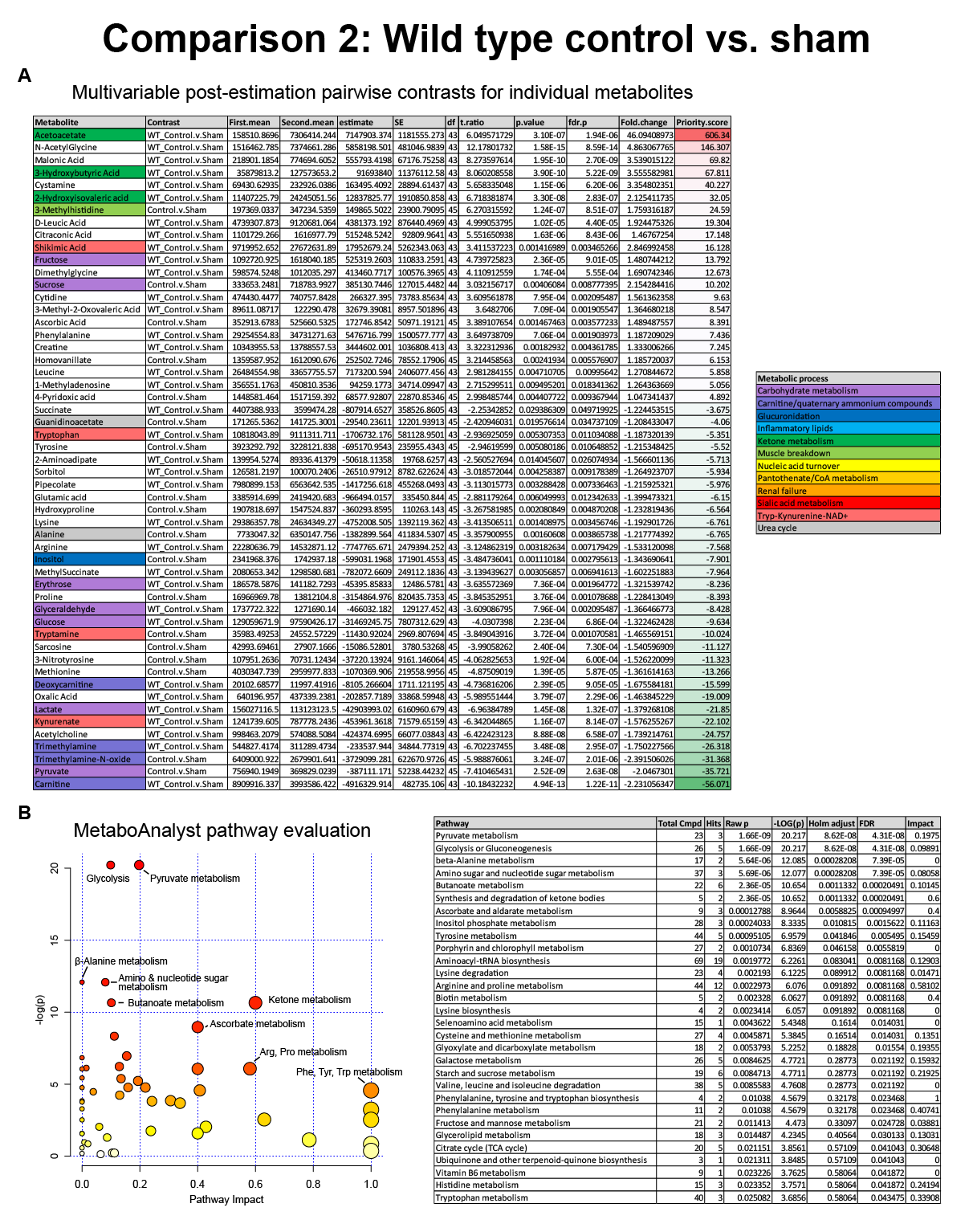


Supplemental Figure 6. Lists of differentially abundant metabolites and altered metabolic pathways between WT and *Ppara^-/-^* mice in the sham condition. A: The most distinctive feature of this comparison is that *Ppara^-/-^* mice have lower levels of ketones than WT mice. Additionally, metabolites associated with tryptophan-kynurenine-NAD^+^ pathway are decreased. Carnitine levels may be elevated in this condition in the *Ppara^-/-^* mice due to inability to utilize fatty acids. B: Pathway analysis using MetaboAnalyst revealed differences in many amino acid pathways, including tryptophan, but also showed altered nicotinamide and TCA cycle metabolism.


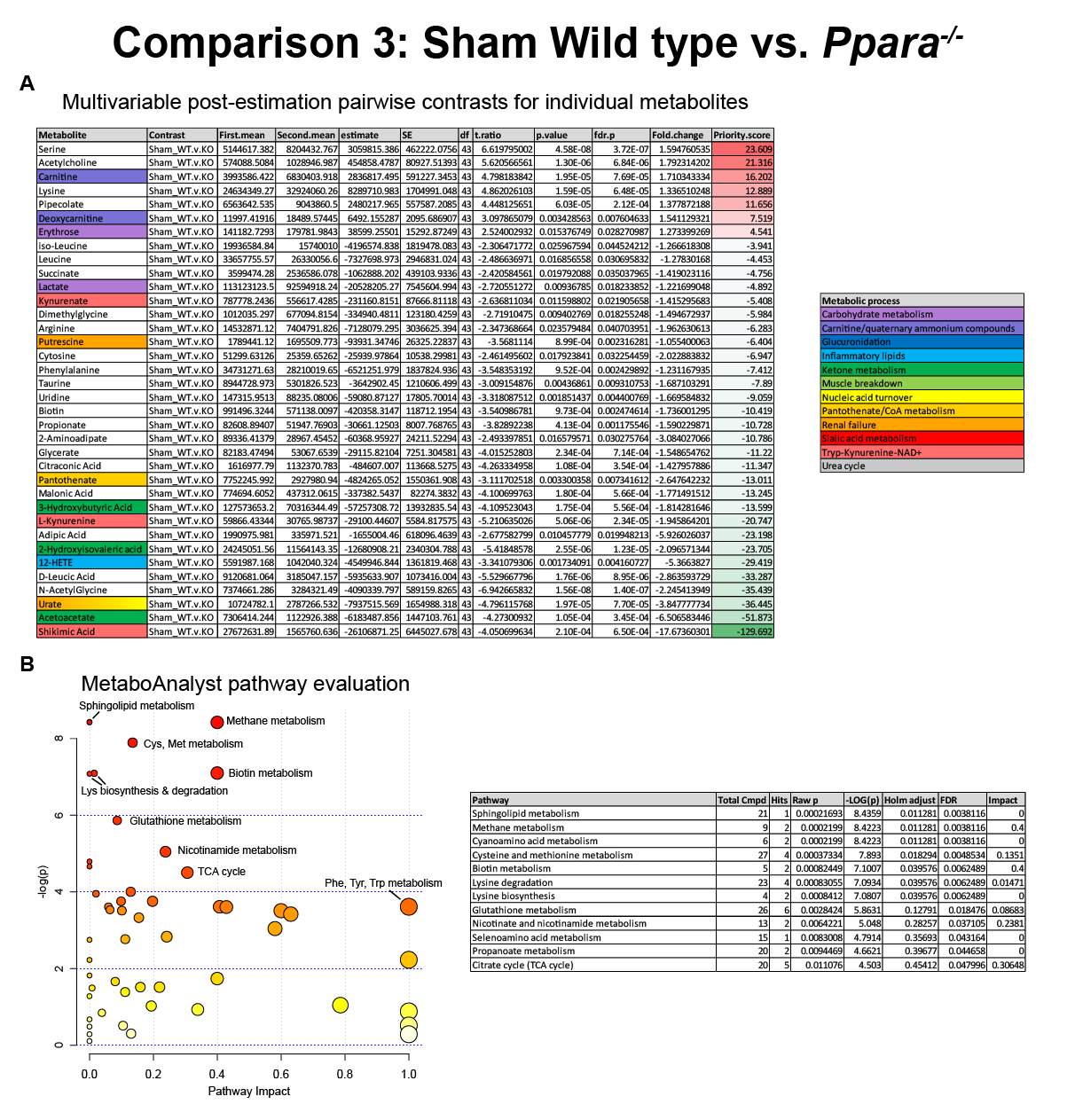


Supplemental Figure 7. Lists of differentially abundant metabolites and altered metabolic pathways between sham and CLP conditions in WT mice. A: Sepsis induces profound metabolic changes, several of which have no current explanation. Levels of glucuronate, N-acetylneuraminate, and pantothenate are remarkably elevated over the sham condition, likely related to stress response mechanisms. Levels of acetoacetate, one of the principle ketones generated in fasting, are lower and metabolites associated with the tryptophan-kynurenine-NAD^+^ pathway are again altered. B: Pathway analysis using MetaboAnalyst revealed a highly significant difference in the β-alanine pathway, which feeds into the pantothenate pathway among others. Also, metabolic pathways involving porphyrin, glucuronate, nicotinamide, ascorbate and various amino acids, including tryptophan, were affected by sepsis.


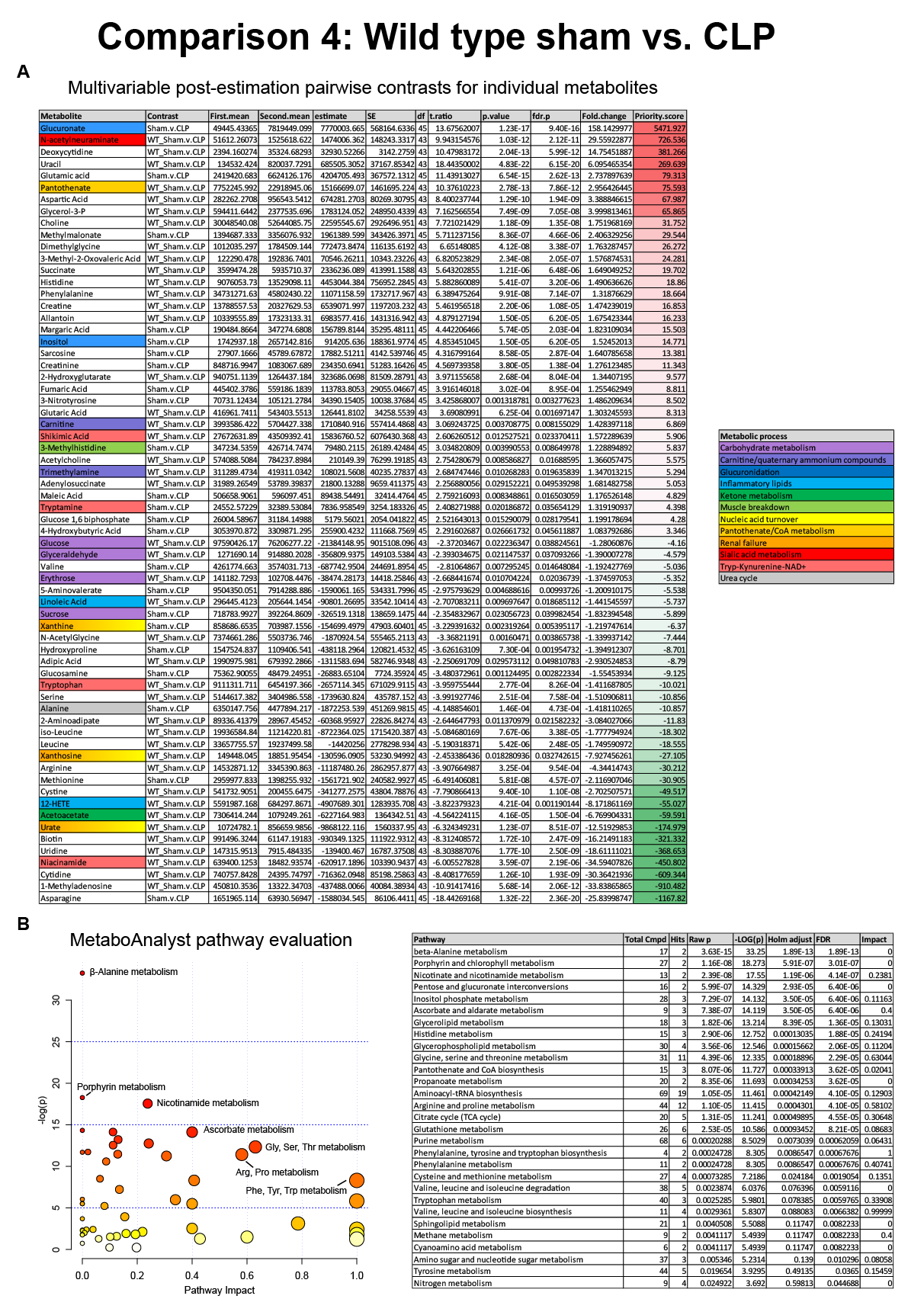


Supplemental Figure 8. PCA analysis and outlier removal from metabolomics data set. A preliminary metabolomics data set was uploaded to MetaboAnalyst to generate a three-dimensional PCA plot. Outliers were identified for subsequent removal from the final data set which then underwent definitive analysis.


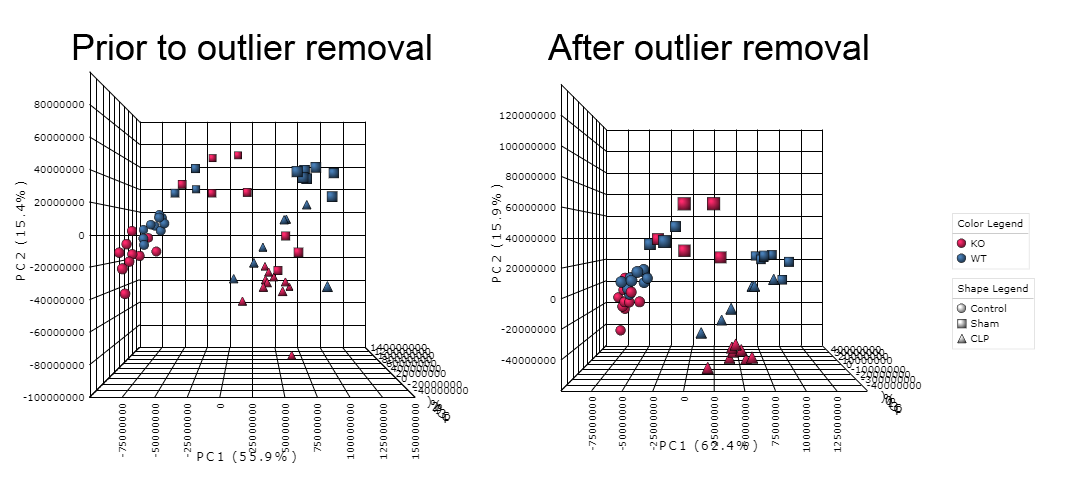


Supplemental Table 1. Liquid chromatography gradient conditions

| Time Segment, min. | Solvent A, % | Solvent B, % |
| --- | --- | --- |
| 0 - 2 | 10 | 90 |
| 2 - 5 | from 10 to 50 | from 90 to 50 |
| 5 - 9 | 50 | 50 |
| 9 - 11 | from 50 to 10 | from 50 to 90 |
| 11 - 20 | 10 | 90 |

Supplemental Table 2. Scoring system for rating proximal tubular injury on electron micrographs.

| **Brush Border** | |
| --- | --- |
| 0 | Intact |
| 1 | Disorganized apical surface, but microvilli intact |
| 2 | Absent/necrotic border |
| **Endocytic vesicles** | |
| 0 | Absent to rare in number. Small. Simple. |
| 1 | Few. Simple. |
| 2 | Moderate in number and size. Simple. |
| 3 | Numerous. Large. Contents more complex. Distributed deep in in to the cell beyond the apical surface. Alternatively, multitude of tiny vacuoles, or absent, in apical necrosis. |
|  |  |
| **Lysosomes** | |
| 0 | Absent to rare. Small. |
| 1 | Few. Small. |
| 2 | Moderate in number and size. |
| 3 | Numerous. Large. Distributed deep in in to the cell beyond the apical surface. Alternatively, absent due to apical necrosis. |
|  |  |
| **Autophagosomes/Autolysosomes** | |
| 0 | Absent |
| 1 | Few in number. Small in size. Simple contents. |
| 2 | Moderate in number, size, and complexity. |
| 3 | Many in number. Large in size. Great in complexity. |
| **Lipid droplets** | |
| 0 | Absent. |
| 1 | Few. |
| 2 | Moderate in number. |
| 3 | Many. |
| **Mitochondria** | |
| 0 | Normal |
| 1 | Slightly disrupted architecture. Some swelling. |
| 2 | Significantly disrupted architecture. |
| 3 | Obliterated. |
| **Interdigitating processes** | |
| 0 | Present |
| 1 | Reduced |
| 2 | Absent |
